# Poor psychosocial work environment: a ticket to retirement? Variations by gender and education

**DOI:** 10.1007/s10433-025-00855-z

**Published:** 2025-04-24

**Authors:** Harpa S. Eyjólfsdóttir, Tale Hellevik, Katharina Herlofson, Axel West Pedersen, Carin Lennartsson, Marijke Veenstra

**Affiliations:** 1 Aging Research Center (ARC), Karolinska Institutet and Stockholm University, Stockholm, Sweden; 2 Centre of Public Health Sciences, Faculty of Medicine, School of Health Sciences, University of Iceland, Reykjavik, Iceland; 3 Norwegian Social Research (NOVA), Oslo Metropolitan University, Oslo, Norway; 4 Swedish Institute for Social Research (SOFI), Stockholm University, Stockholm, Sweden; 5 Health Services Research Unit, Akershus University Hospital, Lørenskog, Norway

**Keywords:** Work exit, Ageing, Psychosocial work characteristics, Job resources, Job strain

## Abstract

Many countries, including Norway, are implementing policies to delay retirement and encourage older workers to remain in the labour market. Improving psychosocial working conditions may motivate older workers to continue working. While research has linked psychosocial working characteristics to retirement intentions and work exit, there is a knowledge gap regarding gender and socioeconomic differences in these influences. This study investigates the impact of psychosocial working characteristics on employment exit among older workers, examining variations by gender and educational attainment. Data were drawn from the Norwegian Life Course, Ageing, and Generation study (NorLAG) collected in 2007 and 2017 (*N* = 2,065) linked to income register data for four subsequent years. Time-to-event analyses revealed that poorer psychosocial working environment increased the likelihood of employment exit. For women, low autonomy was significant, while for men significant associations were found for high job stress, low job variety, lack of appreciation, limited learning opportunities, accumulation of poor job resources, and job strain. Interaction analysis showed only significant gender differences for few learning opportunities and poor job resources. Separate analyses stratified by educational attainment showed no significant association for those with compulsory education, while those with higher levels of education were more likely to retire if faced with low job variety, low autonomy, and poor job resources–yet interaction analysis showed no significant differences. These findings suggest that interventions aimed at delaying retirement should consider gender and socioeconomic differences, providing older workers with more control over their tasks and equitable access to learning opportunities and resources.

**Supplementary Information:**

The online version contains supplementary material available at 10.1007/s10433-025-00855-z.

## Introduction

Implementing pension reforms to encourage longer working lives, and delayed retirement has become a key political priority in response to an ageing population (OECD 2023). However, reforming pension systems is not sufficient; other factors, particularly the psychosocial work environment, are crucial for motivating older workers to remain in the labour market. Previous research has explored the relationship between psychosocial working characteristics and retirement intentions and/or actual work exit (Browne et al. 2019). Some studies have concentrated on adverse or unfavourable psychosocial work characteristics, like low job autonomy or high demands, and how such factors may influence early work exit (e.g. Robroek et al. 2013; Hintsa et al. 2015; Sundstrup et al. 2021). Others have focused on beneficial psychosocial working conditions and their association with later retirement (Virtanen et al. 2014; Stengård et al. 2021; Andersen et al. 2021). Although findings vary, earlier studies tend to indicate a significant relationship between the perceived quality of the psychosocial work environment and preferred or actual timing of labour market exit (e.g. Lund and Villadsen 2005; Harkonmäki et al. 2006; Robroek et al. 2013; Hintsa et al. 2015; Frins et al. 2016; d’Errico et al. 2021; Stengård et al. 2021; Sundstrup et al. 2021). Since psychosocial working conditions are more easily modifiable than several other characteristics related to the timing of retirement (e.g. gender, education, health, economic situation, and caregiving responsibilities (Fisher et al. 2016)), improving these conditions constitutes a promising route to help delay work exits, potentially resulting in considerable public savings.

Despite a large research literature on the association between psychosocial work environment and retirement, few studies have investigated whether the correlation varies across different groups of senior workers. In a systematic review, Browne and colleagues (2019) noted that hardly any studies stratified the analyses by gender or socioeconomic position. Understanding the workplace drivers of retirement behaviour for different groups is valuable, as the finding of heterogeneous effects may indicate the need for tailored interventions. The aim is therefore to investigate the influence of psychosocial working characteristics on exit from paid employment among older workers and to determine whether the influence varies across gender and socioeconomic position (i.e. educational level).

In our analyses, we use nationally representative longitudinal survey data from Norway, collected in 2007 and 2017, linked to annual register data for four subsequent years after each survey wave. The data allow us to follow the same individuals (aged 58 to 65 years at baseline), to observe whether they leave the labour market during the four-year period or remain gainfully employed. This approach enables us to investigate the association between psychosocial work environment and work exit and to determine whether the results vary by gender and educational attainment.

## Background

### Theoretical models

Several theoretical models have been suggested for understanding the association between the psychosocial work environment and work exit (Browne et al. 2019). These models were developed from research on job strain, notably the job demand-control (JDC) model (Karasek 1979), the effort-reward imbalance model ((Siegrist 1996), and the job demands-resources (JD-R) model (Bakker and Demerouti 2007).

The JDC model posits that job strain results from a combination of high demands and low decision latitude, where job demands are psychological stressors related to accomplishing the job tasks, whereas decision latitude refers to workers’ potential control over their tasks and conduct at work (Karasek 1979). Later, workplace social support was integrated into the JDC model (Johnson and Hall 1988; Karasek and Theorell 1990). To complement the JDC(S) model, Siegrist (1996) introduced a model on the (im)balance of efforts and rewards, emphasizing the importance of workplace rewards over job control. The model suggests that a mismatch between the efforts invested, and the rewards received can lead to distress. Finally, the job demands-resources (JD-R) model by Bakker and Demerouti (2007) considers the relation between job demands and job resources. Job resources extend beyond autonomy and control, encompassing various (positive) workplace characteristics that facilitate goal achievement, learning, and personal development, whereas job demands are aspects requiring continuous effort or skills, associated with certain physiological and/or psychological costs. The model proposes that job strain arises when demands are considerable, and resources are scarce.

### Previous research on psychosocial work environment and work exit

The theoretical models outlined above were initially developed to better understand job strain among employees but have proven useful also in retirement studies (e.g. Schreurs et al. 2011; van Solinge and Henkens 2014; Hintsa et al. 2015; Carr et al. 2016; Stengård et al. 2021). The main idea is that high job demands, without sufficient control and/or other resources, may give rise to work overload and strain, potentially leading to (early) retirement. Job resources, on the other hand, are assumed to be associated with job satisfaction and well-being, which may encourage continued labour market participation and later exits, if demands do not become excessive.

Existing research measures the various dimensions of the psychosocial work environment using a range of different conceptualizations and survey items. Browne and colleagues (2019), in their systematic review, categorize positive work characteristics as “job resources”, and include job control, opportunities to develop or learning opportunities, work variety, and recognition at work. They find that job control is by far the most studied resource, but with multiple different operationalizations, such as job autonomy, decision authority, decision latitude, influence at work, and flexibility of work hours/place (Virtanen et al. 2014; ten Have et al. 2015; Carr et al. 2016; Stengård et al. 2021; Andersen et al. 2021). Negative characteristics, i.e. “job demands”, are most often operationalized as work overload or time pressure but cover also aspects like emotional demands and role conflict (Browne et al. 2019).

Browne and colleagues (2019) conclude that the evidence on the relationship between job demands and retirement is inconsistent, whereas job resources, in the form of job control, seem to be essential. Although addressed to a lesser extent in earlier studies, they also find opportunities for self-development to play a significant role. Two recent Scandinavian studies corroborate these findings, suggesting that providing older workers with more control over their work time and learning opportunities, as well as recognition for their achievements, may help retain them in the workforce (Stengård et al. 2021; Andersen et al. 2021). Additionally, Christensen and Knardahl (2022) studying turnover intentions based on data from Norway report that as employee’s age, empowering, and supportive management styles become increasingly important for their willingness to continue working.

The review by Browne et al. (2019) does not include studies addressing psychosocial work aspects and work exits due to disability. However, Knardahl et al. (2017) analysed this association in a review of 20 studies, of which 15 found low job control to contribute to early disability/health-related retirement. Again, evidence regarding the impact of job demands is limited, with only two studies showing a significant association and nine reporting nonsignificant results (Knardahl et al. 2017).

Based on prior research on the importance of job resources and job demands for work exit, we expect our analyses to reveal that older workers who have a more positive experience of their psychosocial work environment–particularly regarding job resources–are more likely to delay their work exit compared to those with a less positive experience.

Our main focus, however, is on whether the association between psychosocial work environment and work exit varies across gender and socioeconomic position. Earlier research shows variations in the quality of the psychosocial work environment across these groups. People holding a lower socioeconomic position (i.e. education, income, and occupational status) tend to have poorer psychosocial working environment compared to those with a higher socioeconomic position (Eyjólfsdóttir et al. 2024). Socioeconomic position reflects different exposures to work-related risks and benefits, and persons with lower levels of education are more often represented in jobs that provide less long-term income security, lower earning potential, and less control over work compared to those with higher education. Evidence so far suggests that the higher educated are more likely to have a better working environment and are in a better position to prolong their working life (Andersen et al., 2020; Eyjólfsdóttir et al. 2024). Not only is the labour market segregated by socioeconomic position, but it also exhibits notable gender segregation, with women disproportionately occupying lower-status jobs and public sector roles in fields like education, health care, and caregiving (Reisel et al. 2019). Consequently, they receive lower wages, often work part-time, and ultimately have lower pensions compared to men (Edge et al. 2017). The predominantly female occupations involve frequent contact with people and meeting other people’s needs; tasks often associated with high psychosocial demands and elevated stress levels (Anxo et al. 2014).

The differences in the psychosocial work environment could be part of the explanation for why men on average retire later than women, and the higher educated later than the lower educated (McAllister et al. 2020). As noted in the introduction, however, there is a lack of studies investigating whether and how these factors influence timing of work exit for men and women respectively or depending on educational level.

Studies exploring possible contrasts by education (or other socioeconomic position indicators) seem to be non-existent, except for Schreurs and colleagues (2011), who address early retirement intentions among blue- and white-collar workers in Belgium. The results reveal different mechanisms for the two groups; high job demands having a stronger direct effect on intended early exit among white-collar workers, and job resources having a more motivating potential for blue-collar workers. Gender differences are, to our knowledge, only addressed specifically in two studies–one from Norway emphasising predictors of early retirement (Blekesaune and Solem 2005), and the other one from Sweden investigating continued labour market participation at age 66–75 years (Farrants et al. 2021). According to the study by Blekesaune and Solem (2005), being employed in an occupation with low autonomy is associated with early retirement, but this effect is observed only for men. The authors assume that differences in work history, which leave women with less financial flexibility to retire early, could be an explanation. In the Swedish study, Farrants et al. (2021) find that low control increases the likelihood of not being employed 11 years later, while high control increases the likelihood of continued employment–regardless of gender. However, for men, the positive correlation between high control and continued employment is only present when job demands are also high. In relation to the last finding, Farrants and colleagues (2021) suggest that jobs with the same level of demands and control could still represent different types of jobs for men and women. They also propose other possible explanations, such as differences in family-work interference, income level, health and morbidity, and social support at work between men and women, but without elaborating further. It should be noted, that in both studies, the authors measure psychosocial working conditions using estimated averages among employees within jobs with the same occupational code and not the study samples’ self-reports, as we rely on in the present study.

Extending this line of research and building on the job demands-resources (JD-R) model, we add to the literature by investigating whether the significance of self-reported psychosocial working conditions for retirement varies by gender and educational attainment. Such knowledge is important in the quest to better understand gender and socioeconomic differences in retirement timing. Furthermore, as psychosocial work factors are modifiable, findings revealing group differences in their effects could be utilized in targeted interventions aimed at raising retirement ages.

Given the limited research and lack of definitive findings in existing studies, we propose two competing hypotheses on how the effects of psychosocial work variables may vary depending on gender and educational level:

Hypothesis 1: The psychosocial work environment will have a *stronger* effect on the timing of work exit for women compared to men, and for individuals with lower educational attainment compared to those with higher educational attainment. This hypothesis is based on the argument that women and the lower educated, due to their relatively weaker labour market position and attachment, may be more sensitive to the quality of their psychosocial work environment, which consequently will have a greater impact on their decision to leave paid employment or continue working.

Hypothesis 2: The psychosocial work environment will have a *weaker* effect on the timing of work exit for women compared to men, and for individuals with lower educational attainment compared to those with higher educational attainment. This hypothesis is based on the argument that women and the lower educated, due to their relatively weaker labour market position and attachment, may be more likely to have other factors overriding the importance of the psychosocial work environment on their decision to leave paid employment or continue working. Such factors may include physically demanding work tasks and health problems inducing early work exits, or insufficient pension accrual delaying work exits. Additionally, women may be more prone to have family considerations influence the timing of their work exits compared to men.

The manner and extent to which the psychosocial work environment can be expected to impact on the timing of work exit will depend on the institutional context, particularly a nation’s retirement regime. As our analyses are based on Norwegian data, a brief overview of Norway’s pension system is needed to contextualize the study.

### Institutional context–the Norwegian retirement regime

In 2011, Norway implemented a major pension reform introducing a more flexible system of claiming pensions combined with stronger incentives to delay labour market exit. Before the reform, old age pensions from the National Insurance System (NIS) could be drawn from age 67 years, with unconditional benefits starting at age 70 years. With the 2011 reform, take-up of NIS old-age pensions can start any time between age 62 years and 75 years with full actuarial adjustment of benefits. Furthermore, old-age pension benefits from NIS can be freely combined with paid employment.

Before 2011, many workers, including all public sector and about half of private sector employees, could retire early under the negotiated early retirement scheme (AFP), allowing withdrawal from age 62 years without actuarial penalties. This arrangement provided strong economic incentives for early retirement. In connection with the pension reform, the AFP scheme for private sector employees was transformed into a life-long supplementary occupational pension scheme with full effect for cohorts born from 1949 onwards, giving instead strong financial incentives for extending working life. In contrast, the public sector maintained the AFP scheme as an early retirement programme, requiring labour market withdrawal for pension access. During the period under study, incentives for prolonging working careers were thus less comprehensive for public sector employees than their private sector counterparts. In addition, several occupational groups in the public sector (e.g. policemen, firefighters, military personnel, and nurses) have occupation-specific early retirement ages. The receipt of disability benefits still serves as an early retirement pathway for workers in both sectors.

Labour market participation rates among older age groups in Norway are comparatively high (OECD 2023). However, less than 50 per cent are gainfully employed when turning 65, and among 67 to 75 year olds, only one out of five are employed (Statistics Norway 2023). The average effective retirement age is around 66 for those employed at age 50 years (NAV 2024). After the pension reform was introduced, there has been a modest increase in older workers’ participation rates, primarily affecting private sector workers, due to the reform’s heterogeneous impact on private and public sector employees (Hernæs et al. 2016; Grødem and Hippe 2021; European Commission 2024).

## Data and methods

### Sample

Our study is a prospective panel study where we combine survey data on perceived psychosocial work environment with public register data to assess the timing of labour market exit. We utilize data from the nationally representative Norwegian Life Course, Ageing, and Generation (NorLAG) study (Veenstra et al. 2021), pooling survey data from wave 2 and wave 3 collected in 2007 and 2017 (hereafter referred to as baseline, with overall response rates 61% and 68%, respectively). These data have been linked to annual income register information for four subsequent years following each survey wave.

**Fig. 1:**
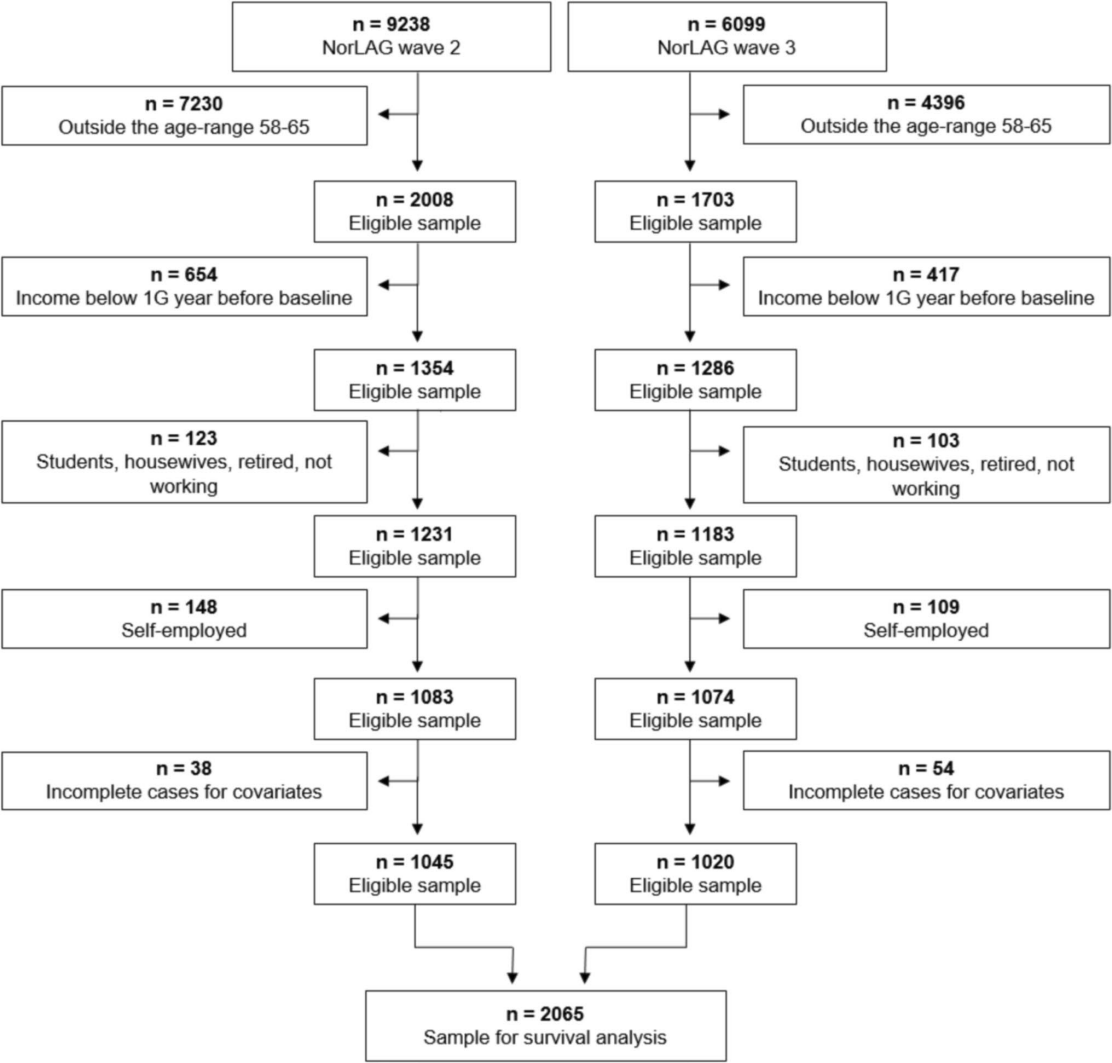
Flowchart

We include employed respondents aged 58–65 years at baseline and follow them for four years, or until age of 66 years, to identify who left and who remains in paid employment at a given year in the follow-up period. The restricted age span was chosen since factors explaining early exits are not necessarily the same as those explaining (very) late exits from the labour market (De Wind et al. 2016; Hellevik and Herlofson 2020), and a broader age range could therefore obscure important effects. Finally, we excluded self-employed respondents from our sample since some of the questions about the psychosocial work environment were only directed at employees. Since the amount of missing due to incomplete cases for covariates was small (n = 92) and not systematic, we decided against multiple imputation and used complete cases in the analyses (total sample N = 2,065, see flowchart Fig. 1).

### Measurements

#### Outcome

We study the influence of perceived psychosocial work environment factors on employment exit among men and women and across different educational levels. To measure the dependent variable, employment exit, we use annual income data from public registries. In each year, a respondent is defined as employed if labour earnings from employment exceed one basic amount (BA) in the Norwegian National Insurance Scheme. A respondent is defined as retired in the year after the last observation of employment, if followed by at least two years of non-employment. The retirement age is the respondent’s age in the last year of employment (Johansson et al. 2016; Eyjólfsdóttir et al. 2021; Hellevik et al. 2023). We do not distinguish between different retirement pathways (i.e. early retirement scheme, old age pension or disability pension) because we are interested in the effect of variation in the psychosocial work environment on work exit in general–irrespective of the specific form it might take.

#### Exposures

Psychosocial work environment is measured at baseline, when everyone in the sample was employed. We assess six individual items measuring different psychosocial work environment characteristics, and construct indexes assessing job resources and job strain (see Table 1 for definitions of each item and for item response distributions). The six items, previously established in Norwegian settings (e.g. Solem 2001; Ipsos 2022), are measured by the questions “To what extent do you in your job experience”: a) a hectic and stressful work situation? b) little variation in tasks (monotonous work)? c) that your management appreciates your work? d) colleagues asking you for advice? e) that you have opportunities for learning new skills? and f) that you have autonomy on the job? Each item had four response categories: 0) to a great extent, 1) to some extent, 2) to a small extent, 3) not at all. For the first two questions, describing a negative psychosocial work aspect, the response categories were reversed so that higher values–for all items–indicate a poorer psychosocial work environment.

**Table 1:** The psychosocial working condition items, response options, and response distributions for the study population (*n* = 2065)

Subject	Wording of questions	Response options and distribution (%)			
	To what extent do you in your work experience:	(0) Not at all	(1) To a small extent	(2) To some extent	(3) To a great extent
Job stress^a^	Hectic and stressful work situation?	8.1	20.4	44.4	27.2
Job variety^a^	Little variation in tasks (monotonous work)?	46.7	29.3	14.7	9.3
		(0) To a great extent	(1) To some extent	(2) To a small extent	(3) Not at all
Appreciated	That your management appreciates your work?	57.2	34.6	6.4	1.8
Give advice	Colleagues asking you for advice?	44.8	44.4	8.6	2.2
Learning opportunities	That you have opportunities for learning new skills?	44.4	38.5	13.7	3.4
Autonomy	That you have autonomy in the job?	57.4	33.2	7.8	1.6
		Dichotomised (%)			
Job resources^b^	Variable showing accumulative poor job resources: Those who had at least two item scores of 2 or higher were added to the poor group	Good resources	Poor resources		
		82.5	17.5		
Job strain^c^	High Stress + Low Autonomy	Low strain	High strain		
		93.5	6.5		

^a^ Items have been reversed

^b^ Job resources were created after a factor analysis of the 6 individual items above. Job variety + Appreciated + Give advice + Learning opportunities + Autonomy loaded on one factor and were added together into a variable with the range of 0–15 and mean 3,3. The variable was dichotomized where those who had at least two item scores of two or higher were added to the Poor resources group

^c^ Low job strain is a combination of having high job stress and low autonomy (decision latitude). It includes people that responded to Job stress “To some extend” or “To a great extent”, and to Autonomy “To a small extend” or “Not at all”

To assess the combined influence of the psychosocial work variables, we did a factor analysis including all six items. The analysis showed that five of these loaded on one dimension, which we call *Job resources*. The exception was experiencing a hectic and stressful work situation—a characteristic typically considered a job demand rather than a job resource (Browne et al. 2019). The five job resource items were summarized into an index ranging from 0 to 15, and then dichotomized where respondents scoring 11 or higher were given the value 1 in the dichotomised variable, indicating an accumulation of poor job resources. Respondents having the two highest values on at least two items were also given the value 1 in the dichotomized variable, indicating an accumulation of poor job resources.

To assess *Job strain*–the combination of high demands and low decision latitude–we combined the following two variables: “hectic and stressful work situation” and “job autonomy”, as suggested by the JD-R model by Bakker and Demerouti (2007). Respondents answering “to some extent” or “to a great extent” to experiencing Job stress, and “to a small extent” or “not at all” to experiencing Autonomy, were categorized as having high job strain (value 1 in the dichotomized (0–1) job strain variable). Online Resource Table S1 shows the correlations between the psychosocial work exposures, including the two constructed indexes.

#### Covariates

The association between psychosocial working conditions and employment exit may be confounded by various conditions, for example age, gender, educational attainment, family situation, working hours, and health (Virtanen et al. 2014; Fisher et al. 2016). We therefore control for important covariates, measured at baseline. These include: age (range 58–65, applied as dummy variables in analysis), gender (0 = men, 1 = women), educational attainment (0 = compulsory, 1 = secondary, 3 = tertiary education), and income (total annual income before taxes in G-units at baseline, in deciles. G is the abbreviation for National Insurance Basic Amount and indicates a basic amount in NOK that the Norwegian Labour and Welfare Administration use to calculate benefits and pensions. This amount is updated every year, i.e. the annual amount corresponded to 33 812 NOK in 2007 and to 93 634 NOK in 2017, and is used here to adjust for inflation and wage growth), (civil status (0 = married/cohabiting, 1 = single), self-rated health (Likert scale from 1–5, with 1 = excellent health and 5 = very poor self-rated health), work hours (0 = part-time, 1 = full-time), sector affiliation (0 = public, 1 = private), and birth cohort (0 = born 1948 or earlier, 1 = born 1949 or later).

#### Statistical analyses

We apply time-to-event analysis to investigate if the quality of the psychosocial work environment at baseline influences subsequent employment exit. We follow the procedure for discrete-time event history analysis, originally suggested by Allison (1984) and elaborated by Jenkins (1995). Following this procedure, we extend the dataset to take the form of person/year units where each respondent contributes with observations up until their employment exit or up to age of 66 years (person-years 8,353, representing 577 employment exits). Participants, who were still working at the end of the four-year follow-up or left the study (due to emigration or death) before leaving work, were treated as right-censored. By transforming the dataset in this way, discrete-time proportional odds can be estimated using logistic regression on the dichotomous outcome variable (Jenkins 2005), indicating whether the individual has retired in the respective year.

To study heterogeneity across the two main sample characteristics (i.e. gender and educational attainment), we employ Pearson’s Chi-squared for binary variables and two sample t-test for continuous variables. We use stepwise logistic regression analysis and present odds ratios (ORs) with 95% confidence interval (95% CI).

In the empirical analyses, we need to consider the fact that the Norwegian retirement regime was significantly changed by the 2011 reform for cohorts born 1949 and onwards, but with rather heterogeneous effects for private sector and public sector employees (Hernæs et al. 2016; European Commission 2024). Hence, all models include an interaction term between employment sector and a variable indicating if the respondents’ birth year was 1949 or later. Analyses were conducted in Stata 17, and figures were created using GraphPad Prism 10.

## Results

### Characteristics of the study population

Sample characteristics are presented in Table 2, and it consists of an approximately equal number of men and women, averaging 61 years old at baseline. Nearly, half have secondary education and 40% have tertiary education. Men have higher total annual income than women. A majority (77%) live with a partner (84% of men and 70% of women). Self-rated health is generally very good for both genders. At baseline, 73% are in full-time employment, with notable gender differences (87% for men and 59% for women). The share between private and public sector is close to equal, but again, with large gender differences (35% of men are employed in the public sector and 69% of women).

**Table 2:** Sample characteristics for total sample, and by men and women separately (%)

	Total	Men	Women	Difference^a^
	*N* = 2,065	*N* = 1,042	*N* = 1,023	*p* value^b^
Age (Mean, SD)	60.84 ± 2.1	60.83 ± 2.1	60.84 ± 2.1	0.93
Educational attainment				0.041
Compulsory	12.3	11.4	13.1	
Secondary	47.6	50.3	44.8	
Tertiary	40.2	38.3	42.1	
Income in G-units (Mean, SD)	7.2 ± 5.8	8.7 ± 7.6	5.7 ± 2.4	< 0.001
Living with partner				< 0.001
No	23.0	16.1	29.9	
Yes	77.0	83.9	70.1	
Self-rated health (Range 1–5, Mean, SD)	1.5 ± 1.1	1.5 ± 1.1	1.5 ± 1.1	0.90
Working part-time or full-time				< 0.001
Part-time	26.9	13.4	40.6	
Full-time	73.1	86.6	59.4	
Private or public sector				< 0.001
Public	51.5	34.6	68.7	
Private	48.5	65.4	31.3	
Birth cohort				0.88
Born in 1948 or earlier	45.0	44.8	45.2	
Born in 1949 or later	55.0	55.2	54.8	
Employment exit				0.001
No	72.1	75.2	68.8	
Yes	27.9	24.8	31.2	

Note: Data are presented as % for categorical measures, and mean ± SD for continuous measures. Income in G-units is here presented as a continuous measure but is used in deciles in regression analysis because of skewness

^a^Significance test of gender differences in sample characteristics

^b^Pearson’s Chi-squared for binary and categorical variables, and two sample t-test for continuous variables

Table 3 presents the distribution of employment exit and psychosocial work exposures by gender and educational attainment. During the 4-year follow-up, 28% left paid employment before reaching age of 67 years, with women exiting at an average age of 63 years, one year earlier than men. Compared to men, women generally feel less appreciated by their supervisor, are less frequently consulted by colleagues, have fewer learning opportunities, experience less job autonomy, and report higher job strain. We also find significant differences based on educational attainment; those with tertiary education tend to leave a year later than those with lower educational attainment. Moreover, people with lower education report poorer psychosocial working environment compared to the higher educated. Online Resource Figure S1 shows the Kaplan–Meier curves with number at risk and number of failures for the whole sample, for gender and for educational attainment. Actual survival was calculated using the Kaplan–Meier method and compared using the log-rank test for gender and cox regression for educational attainment. Online Resource Figure S2 illustrates the average level of psychosocial work exposures at each age of employment exit and Online Resource Figure S3 shows the average age of employment exit over job resources and job strain.

**Table 3:** Average of age of employment exit and the distribution of psychosocial work exposures over gender and educational attainment (*n* = 2065)

		Gender			Educational attainment			
	Total	Men	Women	*p*-value^a^	Compulsory	Secondary	Tertiary	*p*-value^a^
	*N* = 2,065	*N* = 1,042	*N* = 1,023		*N* = 253	*N* = 982	*N* = 830	
Employment exit (n, %)								
No	1,488 (72.1)	784 (75.2)	704 (68.8)	0.001	182 (71.9)	680 (69.2)	626 (75.4)	0.014
Yes	577 (27.9)	258 (24.8)	319 (31.2)		71 (28.1)	302 (30.8)	204 (24.6)	
Age of employment exit (Range 58–66, Mean ± SD)								
	63.41 ± 1.7	63.53 ± 1.6	63.30 ± 1.7	0.099	63.33 ± 1.8	63.18 ± 1.6	63.76 ± .1.6	< 0.001
Psychosocial work exposures (Range 0–3, Mean ± SD)								
Job stress	1.9 ± .87	1.9 ± .86	1.9 ± .87	0.300	1.8 ± .95	1.9 ± .88	1.9 ± .82	0.032
Job variety	0.9 ± .97	0.9 ± .98	0.9 ± .96	0.130	1.2 ± 1.10	1 ± 1.00	0.7 ± .85	< 0.001
Appreciated	0.5 ± .67	0.4 ± .65	0.5 ± .70	0.036	0.5 ± .71	0.5 ± .68	0.4 ± .64	0.100
Give advice	0.7 ± .74	0.6 ± .71	0.8 ± .76	< 0.001	1 ± .84	0.7 ± .75	0.5 ± .63	< 0.001
Learning opportunities	0.7 ± .81	0.7 ± .80	0.8 ± .82	0.014	1 ± .97	0.8 ± .83	0.6 ± .69	< 0.001
Autonomy	0.5 ± .70	0.4 ± .66	0.6 ± .73	< 0.001	0.6 ± .81	0.5 ± .74	0.4 ± .61	< 0.001
Job resources (n, %)				0.260				< 0.001
Good job resources	1,703 (82.5)	869 (83.4)	834 (81.5)		176 (69.6)	774 (78.8)	753 (90.7)	
Poor job resources	362 (17.5)	173 (16.6)	189 (18.5)		77 (30.4)	208 (21.2)	77 (9.3)	
Job strain (n, %)				0.031				0.002
Low strain	1,930 (93.5)	986 (94.6)	944 (92.3)		228 (90.1)	908 (92.5)	794 (95.7)	
High strain	135 (6.5)	56 (5.4)	79 (7.7)		25 (9.9)	74 (7.5)	36 (4.3)	

Higher scores reflect poorer psychosocial working environment

Data are presented as mean ± SD for continuous measures, and n (%) for categorical measures

^a^Significance tests: Pearson’s Chi-squared for binary variables and two sample t-test for continuous variables

#### Psychosocial working conditions and employment exit

**Fig. 2:**
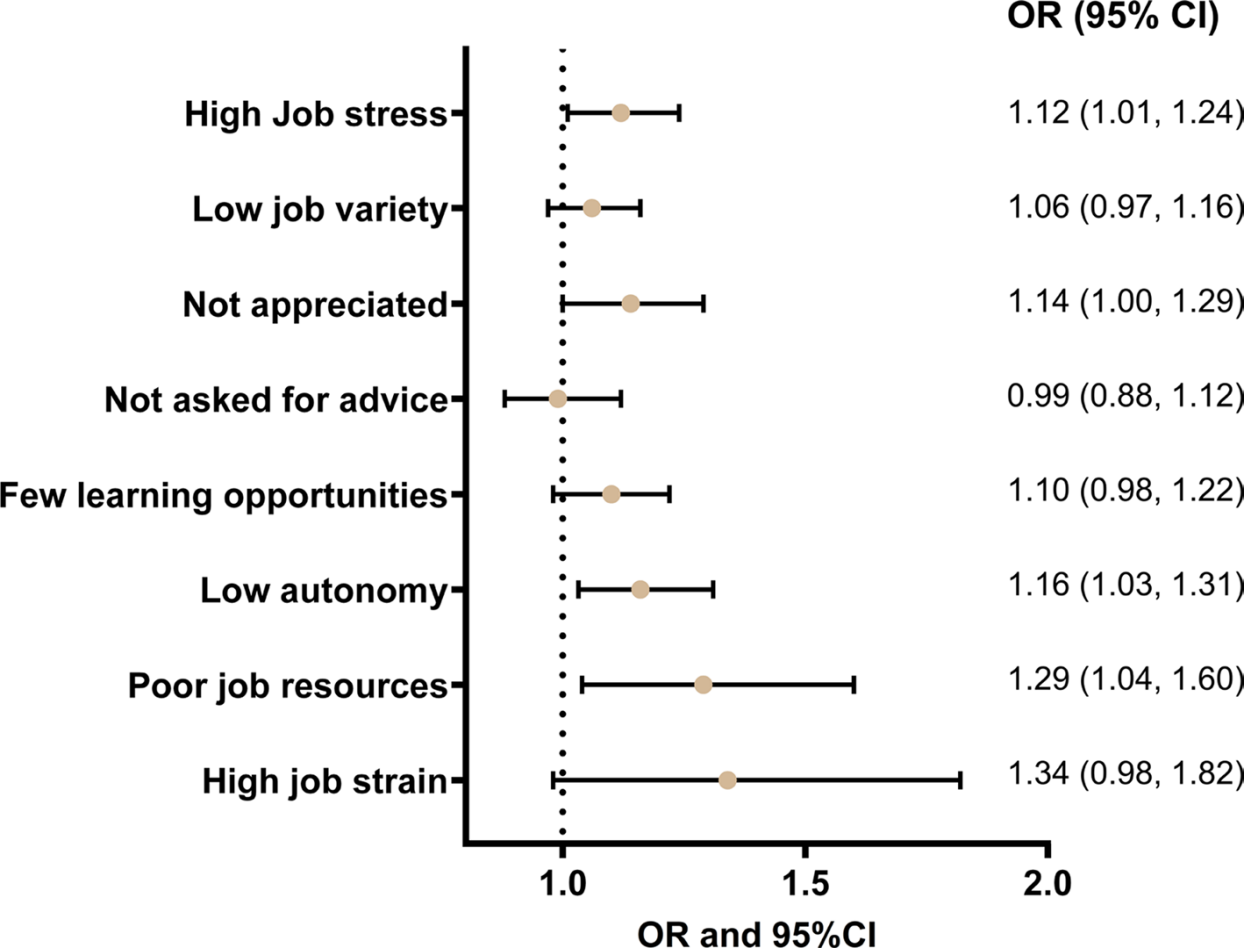
Results from logistic regression analysis showing the association of the eight psychosocial job exposures with employment exit, respectively. Note: Adjusted for age, gender, educational attainment, income, civil status, self-rated health, working part-time of full-time, and including an interaction between employment sector and birth year 1948 or earlier. Models are not mutually adjusted for psychosocial job exposures

To study the probability of employment exit (the dependent variable) during follow-up, we used logistic regression analysis. The impacts of each single psychosocial working environment exposure and the two indexes, adjusted for all covariates (age, gender, educational attainment, income, civil status, self-rated health, working full-time or part-time, birth cohort, and employment sector), are summarized in Fig. 2 (Online Resource Table S2 includes the results also for all covariates). The analyses show that every unit increase of job stress (OR 1.12, 95% CI 1.01–1.24), not feeling appreciated (OR 1.14, 95% CI 1.00–1.29), having low job autonomy (OR 1.16, 95% CI 1.03–1.31), and experiencing an accumulation of poor job resources (OR 1.29, 95% CI 1.04–1.60) all significantly increase the odds of employment exit in a particular year during the 4-year follow-up (Fig. 2). The results also show that those who are older, have higher education, are married/cohabitating, and have poorer self-rated health are more likely to leave employment (Online Resource Table S2).

#### Psychosocial working conditions and employment exit by gender

**Fig. 3:**
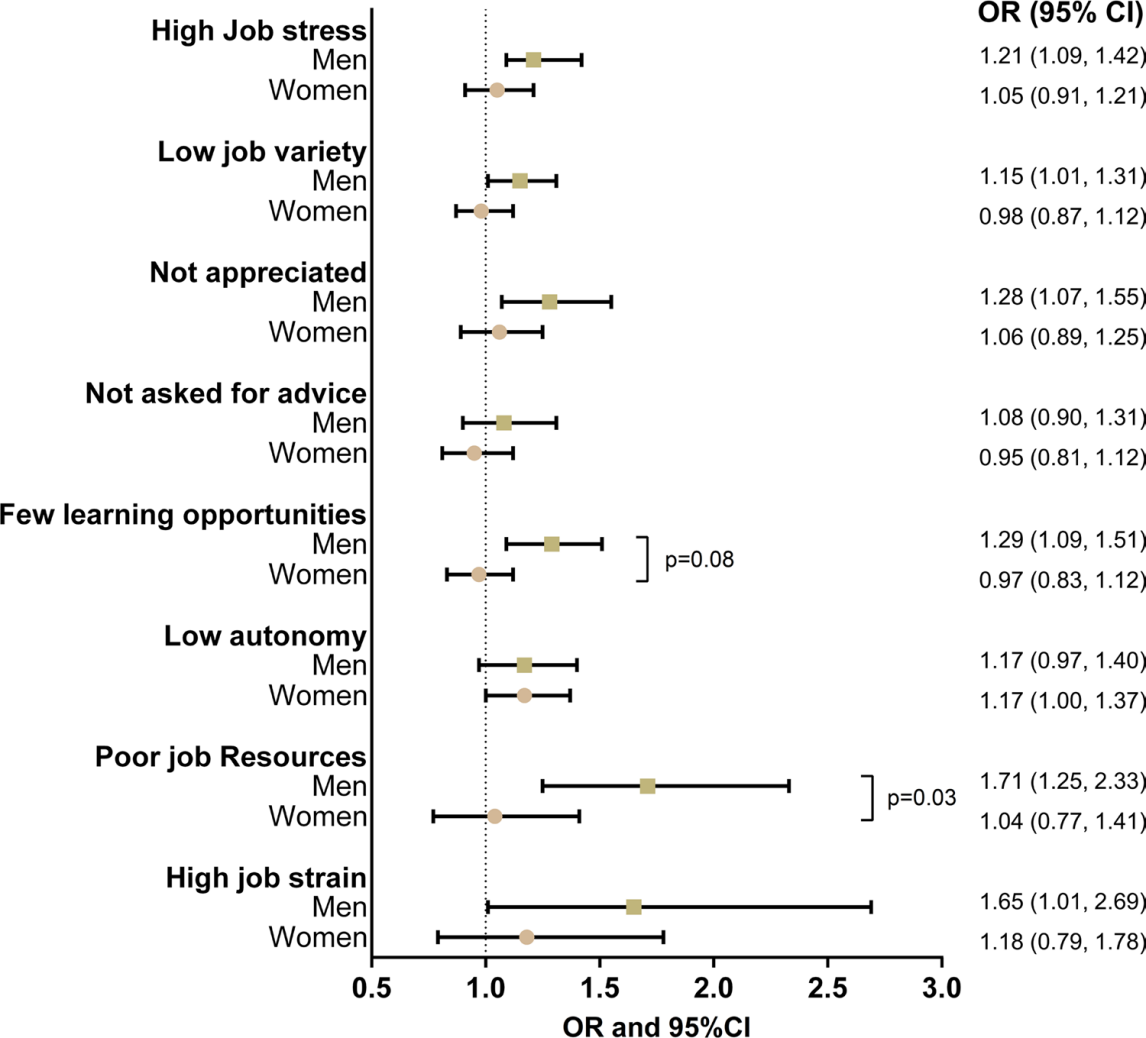
Results from logistic regression analysis showing the association of the eight psychosocial job exposures with employment exit for men and women. Note: Adjusted for age, educational attainment, income, civil status, self-rated health, working part-time of full-time, and including an interaction between employment sector and birth year being in 1948 or earlier. Models are not mutually adjusted for other psychosocial job exposures. ] indicates a significant interaction, and the *p*-value for the interaction terms is displayed

To investigate whether the associations between psychosocial working environment and employment exit vary by gender, we conducted separate logistic regression analyses for men and women. The results from the fully adjusted models are presented in Fig. 3. For women, only having low autonomy significantly increased the odds of employment exit. For men, on the other hand, experiencing high job stress (OR 1.21, 95% CI 1.09–1.42), low job variety (OR 1.15, 95% CI 1.01–1.31), not feeling appreciated (OR 1.28, 95% CI 1.07–1.55), few learning opportunities (OR 1.29, 95% CI 1.09–1.51), accumulation of poor job resources (OR 1.71, 95% CI 1.25–2.33) and job strain (OR 1.65, 95% CI 1.01–2.69) all significantly increased the risk of employment exit during follow-up. Interaction analyses revealed statistically significant gender differences only for few learning opportunities and the index of accumulation of poor job resources, suggesting a stronger impact on employment exit for men compared to women, when controlling for age, gender, educational attainment, income, civil status, self-rated health, working full-time or part-time, birth cohort, and employment sector.

#### Psychosocial working conditions and employment exit by educational attainment

**Fig. 4:**
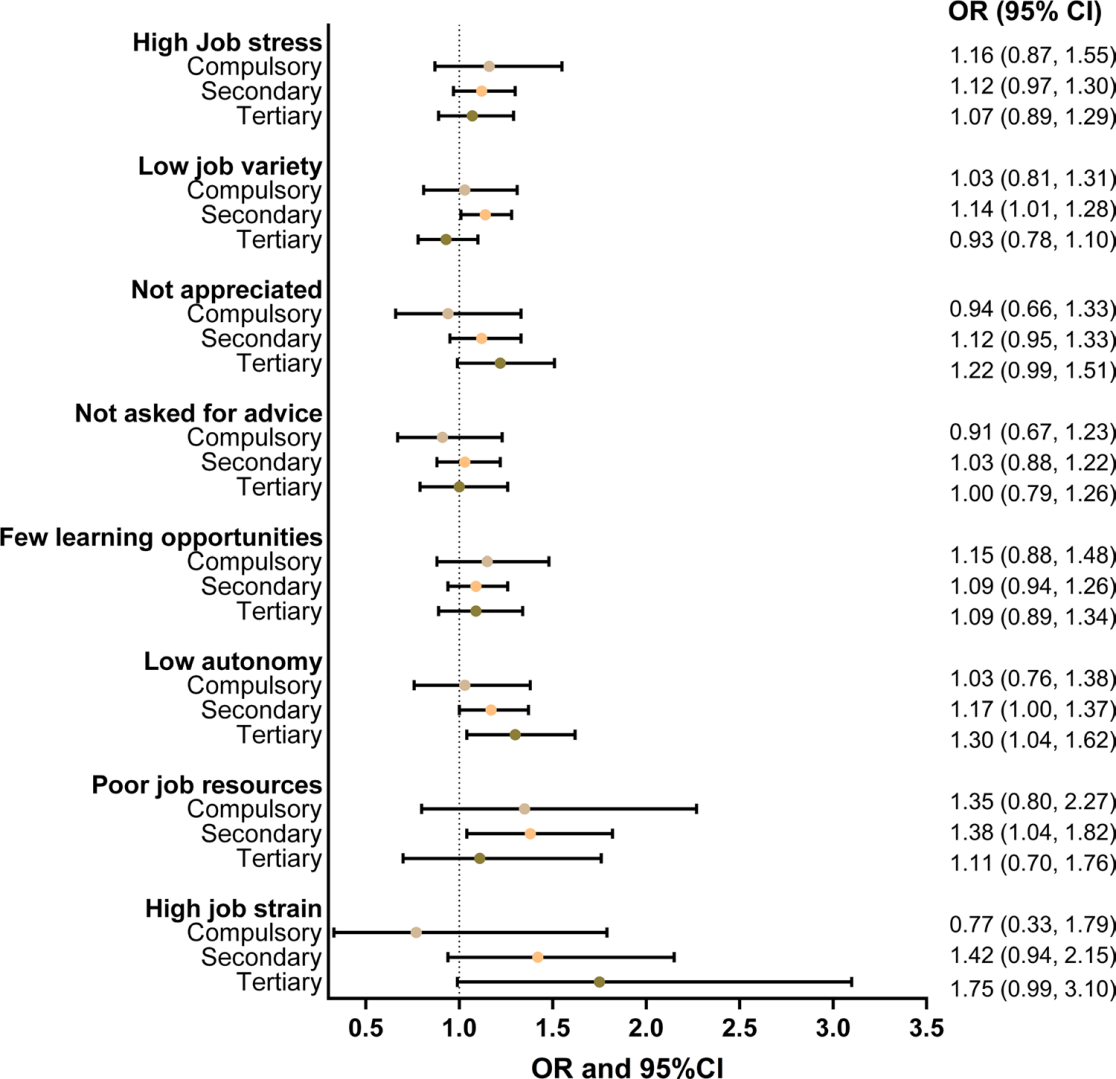
Results from logistic regression analysis showing the association of the eight psychosocial job exposures with employment exit by educational attainment. Note: Adjusted for age, gender, income, civil status, self-rated health, working part-time of full-time, and including an interaction between employment sector and birth year being in 1948 or earlier. Models are not mutually adjusted for other psychosocial job exposures

Next, we explored whether psychosocial working conditions influence employment exit differently by educational attainment by conducting separate logistic regression analyses for the three educational levels. Figure 4 shows the results from the fully adjusted models for each psychosocial working environment exposure. For employees with compulsory education, none of the psychosocial job exposures are statistically significantly associated with employment exit. For those with secondary education on the other hand, the odds of employment exit are significantly increased if they experience low job variety (OR 1.14, 95% CI 1.01–1.28), low autonomy (OR 1.17, 95% CI 1.00–1.37), and an accumulation of poor job resources (OR 1.38, 95% CI 1.04–1.82). Finally, for people with tertiary education, low autonomy (OR 1.30, 95% CI 1.04–1.62) significantly increases the odds of employment exit, and—without reaching statistical significance—high job strain (OR 1.75, 95% CI 0.99–3.10) as well. Interaction analyses showed, however, no significant interactions between the eight exposures and educational attainment, respectively, implying that the association between the psychosocial environment items and work exit does not differ significantly across the different educational levels.

## Discussion

Our study, based on data from Norway, supports previous research regarding the impact of psychosocial work environment on subsequent work exit: perceived job resources do make a difference for when older workers leave paid employment (Browne et al. 2019; Stengård et al. 2021; Andersen et al. 2021). In line with the job demands-resources (JD-R) model, both specific exposures (i.e. low job autonomy, lack of learning opportunities and not feeling appreciated by the management) and the accumulation of poor job resources, as well as job strain, increase the odds of exiting paid employment within the four subsequent years. However, the strength of the associations varies across different groups of older workers, implying that while a poor psychosocial environment at the workplace might be a ticket to retirement for some, it is not necessarily the case for everyone.

Starting with gender, our analyses revealed clear differences in the perceived psychosocial work environment, with women generally experiencing less favourable conditions than men. Women were also more likely to leave paid employment in the follow-up period. Nonetheless, fully adjusted models showed that only low job autonomy was significantly associated with work exit for women. Men, on the other hand, had increased odds of leaving paid employment if they experienced high job stress, low job variety, felt unappreciated, lacked learning opportunities, had accumulated poor resources, or experienced job strain. The final interaction analyses, however, revealed that only with respect to the absence of learning opportunities and the accumulation of poor job resources, were the associations significantly stronger for men than for women, providing support for H2. These findings align with the JD-R model and corroborate previous research, which demonstrates that opportunities for self-development may aid in retaining individuals within the workforce, though our findings apply only to men and not women. This offers a theoretical contribution to the JD-R model by suggesting a gender-specific impact of job resources on employment exit.

Regarding educational attainment, the group with the lowest educational level reported the poorest psychosocial working environment, and they were also more likely to exit paid employment in the follow-up period. However, the association between psychosocial work characteristics and subsequent employment exit was not significant for the lowest educated. Employees with secondary education, on the other hand, were more inclined to leave paid employment if they experienced low job variety and poor job resources. Finally, the higher educated, i.e. employees with tertiary education, reported a more positive psychosocial work environment on average, but when facing low autonomy and high job strain, they were more likely to retire. Yet, the interaction analyses showed no significant differences across educational levels in the associations between perceived psychosocial work environment and exit from paid employment, leading us to reject both H1 and H2.

The relative insensitivity to psychosocial working conditions observed among women—autonomy being the only exception—could be an indication that their retirement is less voluntary and more driven by forcing factors, either in the direction of early or late retirement. For example, women are more likely than men to have jobs with occupation-specific early retirement ages. Moreover, they tend to align their retirement with that of their male partners (Syse et al. 2014) and are also more likely than men to face conflicting caregiving responsibilities (Herlofson and Brandt 2020), which may drive them to leave the labour market regardless of their (dis)satisfaction with the psychosocial work environment. Conversely, because women’s careers more frequently involve leaves of absence and part-time work compared to men’s, they may need to continue working, regardless of their perception of psychosocial work factors, to attain adequate pension accrual.

In a similar manner, the stratified analyses showed that psychosocial working conditions were not significant factors for work exit among the lower educated. Workers with low educational attainment tend to be overrepresented in more precarious, lower-paying, manual jobs. For these employees, straining physical working conditions might be more important triggers for early retirement than psychosocial conditions. On the other hand, working in low-paying jobs could also force some of them to postpone retirement due to insufficient pension accrual, even in the face of adverse psychosocial working conditions.

The fact that both women and low educated groups tend to retire early, irrespective of the quality of the psychological work environment, could reinforce the growing concern that a pension system, which emphasizes individual choice and provides strong economic incentives for postponing retirement, may exacerbate social inequalities in old age. According to two recent studies by Halvorsen and Pedersen (2022) and Hernæs et al. (2024), there are indications that the Norwegian pension system could become regressive due to the social gradient in the propensity to take advantage of the economic incentives offered to workers who postpone retirement.

### Strengths and limitations

This study combines survey data on the psychosocial work environment with annual public registry income data. By examining various psychosocial work characteristics and analysing the results by gender and education, our study provides a more nuanced understanding of how the psychosocial work environment influences employment exit. It thereby contributes theoretically to the JD-R model by demonstrating that the impact of job resources may vary across different groups. Socioeconomic position was assessed though educational attainment, but the models were also adjusted for total annual income at baseline. Future research should assess heterogeneity across additional social groups, for example considering other socioeconomic indicators such as material deprivation, wealth, or occupational class, to further inform sustainable policies for extending working lives.

Assessing work exit through register data minimizes subjective recall errors (Eyjólfsdóttir et al. 2021; Hellevik et al. 2023), but it does not provide the exact date of retirement. Still, it allows us to determine the year and age of retirement. A four-year follow-up enhances our confidence in correctly estimating employment exits, although this design may limit our ability to fully assess the psychosocial work environment’s impact, as the time between baseline interviews and the observed exits varies among the respondents, including interaction terms between the different exposures and time elapsed before employment exit, did, however, not unveil any duration dependency. Furthermore, we incorporate two waves of data collected a decade apart, during which a pension reform was implemented that could potentially introduce systematic differences. We conducted stratified analysis for the two waves which showed no significant differences in the association between psychosocial work exposures and employment exit; we are thus confident in merging the data together. Results are available upon request to the corresponding author.

## Conclusion

As populations are ageing, most developed countries are raising retirement ages to enhance the fiscal sustainability of welfare and pension systems (European Commission 2021). Our study confirms that a poor psychosocial work environment increases the risk of employment exit, indicating that improving the psychosocial conditions at work could indeed delay retirement. We found this to be particularly the case for men and the higher educated, pointing towards an added value if legislators, organizations, and employers consider gender and socioeconomic differences when designing workplace policies and programmes. While our findings suggest that improvements in psychosocial work characteristics may not equally delay retirement across all groups, an overall better psychosocial environment at Norwegian workplaces will still benefit all employees, regardless of their age, career stage, gender, or educational attainment.

## Supplementary Information

Below is the link to the electronic supplementary material.

Supplementary file1 (DOCX 474 KB)

## Data Availability

Publicly available datasets were analysed in this study. This data can be found here: https://norlag.nsd.no/.
