# Supplementary material for: Poor psychosocial work environment: a ticket to retirement? Variations by gender and education
Source: Eur J Ageing. 2025 Apr 24;22(1):18. doi: 10.1007/s10433-025-00855-z (PMC12022190; doi:10.1007/s10433-025-00855-z)
Supplement: Supplementary file 1 — Supplementary file1 (DOCX 474 KB) [file 10433_2025_855_MOESM1_ESM.docx]

**Supplementary Information**

**Poor psychosocial work environment – a ticket to retirement? Variations by gender and education**

*European Journal of Ageing*

Harpa S. Eyjólfsdóttir, PhD^1,2*^

Tale Hellevik, PhD^3^

Katharina Herlofson, PhD^3^

Axel West Pedersen, PhD^3^

Carin Lennartsson, PhD^2,4^

Marijke Veenstra, PhD^5^

^1^ Aging Research Center (ARC), Karolinska Institutet and Stockholm University, Stockholm, Sweden

^2^ Centre of Public Health Sciences, Faculty of Medicine, School of Health Sciences, University of Iceland, Reykjavik, Iceland

^3^ Norwegian Social Research (NOVA), Oslo Metropolitan University, Oslo, Norway

^4^ Swedish Institute for Social Research (SOFI), Stockholm University, Sweden

^5^ Health Services Research Unit, Akershus University Hospital, Lørenskog, Norway

**Correspondence to:**

Harpa S. Eyjólfsdóttir, PhD

Aging Research Center (ARC), Karolinska Institutet and Stockholm University, Stockholm, Sweden

Tomtebodavägen 18A, 17165 Solna, Sweden

Tel: +354 865 18 39

Email: [harpa.eyjolfsdottir@ki.se](mailto:harpa.eyjolfsdottir@ki.se)
ORCID: 0000-0003-1478-5550

| Supplementary Table S1: Correlation between psychosocial work exposures | | | | | | | | | |
| --- | --- | --- | --- | --- | --- | --- | --- | --- | --- |
|  | High job stress | Low job variety | Not appreciated | Not asked for advice | Few learning opportunities | Low autonomy | Poor job resources | High job strain |  |
| High job stress | 1 |  |  |  |  |  |  |  |  |
| Low job variety | 0,00 | 1 |  |  |  |  |  |  |  |
| Not appreciated | 0,04 | 0,16 | 1 |  |  |  |  |  |  |
| Not asked for advice | -0,14 | 0,21 | 0,23 | 1 |  |  |  |  |  |
| Few learning opportunities | 0,01 | 0,26 | 0,36 | 0,35 | 1 |  |  |  |  |
| Low autonomy | 0,07 | 0,20 | 0,29 | 0,22 | 0,28 | 1 |  |  |  |
| Poor job resources | -0,04 | 0,42 | 0,38 | 0,44 | 0,53 | 0,39 | 1 |  |  |
| High job strain | 0,16 | 0,12 | 0,19 | 0,09 | 0,21 | 0,62 | 0,20 | 1 |  |

| Supplementary Table S2. Logistic regressions showing the Odds Ratio (OR) and 95% Confidence Interval (95%CI) of employment exit by the eight exposure variables and all control variables, separately (n=8,353) | | | | | | | | |
| --- | --- | --- | --- | --- | --- | --- | --- | --- |
|  | **High job stress** | **Low job variety** | **Not appreciated** | **Not asked for advice** | **Few learning opportunities** | **Low autonomy** | **Poor job resources** | **High job strain** |
|  | **OR (95%CI)** | **OR (95%CI)** | **OR (95%CI)** | **OR (95%CI)** | **OR (95%CI)** | **OR (95%CI)** | **OR (95%CI)** | **OR (95%CI)** |
| Exposure |  |  |  |  |  |  |  |  |
| Good psychosocial environment | Reference group |  |  |  |  |  |  |  |
| Poor psychosocial environment | 1,12 (1,01, 1,24) | 1,06 (0,97, 1,16) | 1,14 (1,00, 1,29) | 0,99 (0,88, 1,12) | 1,10 (0,98, 1,22) | 1,16 (1,03, 1,31) | 1,29 (1,04, 1,60) | 1,34 (0,98, 1,82) |
| Age, dummies |  |  |  |  |  |  |  |  |
| 58 | Reference group |  |  |  |  |  |  |  |
| 59 | 1,13 (0,35, 3,72) | 1,14 (0,35, 3,73) | 1,14 (0,35, 3,73) | 1,14 (0,35, 3,73) | 1,14 (0,35, 3,72) | 1,15 (0,35, 3,78) | 1,14 (0,35, 3,72) | 1,15 (0,35, 3,76) |
| 60 | 1,60 (0,54, 4,75) | 0,54 (4,75, 1,60) | 1,60 (0,54, 4,75) | 1,60 (0,54, 4,75) | 1,60 (0,54, 4,76) | 1,62 (0,54, 4,80) | 1,59 (0,54, 4,73) | 1,61 (0,54, 4,78) |
| 61 | 5,93 (2,15, 16,32) | 2,15 (16,32, 5,92) | 5,92 (2,15, 16,29) | 5,92 (2,15, 16,28) | 5,92 (2,15, 16,3) | 5,98 (2,17, 16,47) | 5,88 (2,14, 16,19) | 5,97 (2,17, 16,43) |
| 62 | 9,74 (3,57, 26,56) | 3,57 (26,56, 9,67) | 9,72 (3,57, 26,52) | 9,67 (3,55, 26,37) | 9,71 (3,56, 26,47) | 9,82 (3,6, 26,77) | 9,63 (3,53, 26,26) | 9,79 (3,59, 26,71) |
| 63 | 6,93 (2,52, 19,08) | 2,52 (19,08, 6,83) | 6,88 (2,5, 18,95) | 6,83 (2,48, 18,81) | 6,86 (2,49, 18,9) | 6,98 (2,53, 19,21) | 6,81 (2,47, 18,74) | 6,95 (2,53, 19,15) |
| 64 | 9,35 (3,40, 25,71) | 3,40 (25,71, 9,2) | 9,29 (3,38, 25,52) | 9,2 (3,35, 25,27) | 9,25 (3,37, 25,42) | 9,41 (3,43, 25,87) | 9,16 (3,33, 25,18) | 9,37 (3,41, 25,76) |
| 65 | 12,4 (4,51, 34,08) | 4,51 (34,08, 12,2) | 12,25 (4,46, 33,7) | 12,17 (4,43, 33,5) | 12,2 (4,44, 33,51) | 12,49 (4,54, 34,3) | 12,10 (4,4, 33,26) | 12,46 (4,53, 34,3) |
| 66 | 3,06 (1,02, 9,16) | 1,02 (9,16, 3,00) | 3,03 (1,01, 9,07) | 3,00 (1,00, 8,98) | 3,01 (1,00, 8,99) | 3,10 (1,03, 9,27) | 2,99 (1,00, 8,95) | 3,07 (1,03, 9,20) |
| Sex |  |  |  |  |  |  |  |  |
| Men | Reference group |  |  |  |  |  |  |  |
| Women | 0,96 (0,78, 1,19) | 1 (0,82, 1,23) | 0,99 (0,81, 1,21) | 0,99 (0,81, 1,22) | 1 (0,81, 1,22) | 0,98 (0,8, 1,21) | 1 (0,82, 1,23) | 0,99 (0,81, 1,21) |
| Education |  |  |  |  |  |  |  |  |
| Compulsory | Reference group |  |  |  |  |  |  |  |
| Secondary | 1,40 (1,06, 1,85) | 1,42 (1,08, 1,89) | 1,42 (1,07, 1,88) | 1,41 (1,07, 1,87) | 1,43 (1,08, 1,89) | 1,42 (1,07, 1,88) | 1,43 (1,08, 1,9) | 1,43 (1,08, 1,89) |
| Tertiary | 1,39 (1,02, 1,91) | 1,44 (1,05, 1,97) | 1,42 (1,04, 1,95) | 1,41 (1,03, 1,94) | 1,44 (1,05, 1,97) | 1,43 (1,04, 1,95) | 1,46 (1,06, 2,00) | 1,42 (1,04, 1,95) |
| Income (deciles) |  |  |  |  |  |  |  |  |
| 1 | Reference group |  |  |  |  |  |  |  |
| 2 | 0,86 (0,62, 1,19) | 0,89 (0,64, 1,23) | 0,88 (0,63, 1,22) | 0,88 (0,63, 1,22) | 0,88 (0,64, 1,23) | 0,89 (0,64, 1,23) | 0,91 (0,65, 1,27) | 0,88 (0,64, 1,23) |
| 3 | 0,77 (0,54, 1,11) | 0,78 (0,55, 1,12) | 0,78 (0,55, 1,12) | 0,78 (0,55, 1,12) | 0,8 (0,56, 1,15) | 0,78 (0,54, 1,12) | 0,8 (0,56, 1,14) | 0,78 (0,55, 1,12) |
| 4 | 0,69 (0,48, 1) | 0,72 (0,5, 1,03) | 0,72 (0,5, 1,04) | 0,71 (0,49, 1,03) | 0,73 (0,51, 1,06) | 0,73 (0,51, 1,05) | 0,74 (0,52, 1,07) | 0,72 (0,5, 1,03) |
| 5 | 0,71 (0,49, 1,02) | 0,72 (0,5, 1,04) | 0,72 (0,5, 1,04) | 0,72 (0,5, 1,03) | 0,73 (0,51, 1,05) | 0,73 (0,51, 1,05) | 0,74 (0,51, 1,07) | 0,72 (0,5, 1,04) |
| 6 | 0,54 (0,35, 0,82) | 0,55 (0,36, 0,84) | 0,55 (0,36, 0,84) | 0,55 (0,36, 0,83) | 0,56 (0,37, 0,86) | 0,56 (0,37, 0,85) | 0,57 (0,37, 0,87) | 0,55 (0,36, 0,84) |
| 7 | 0,46 (0,3, 0,7) | 0,47 (0,31, 0,72) | 0,47 (0,31, 0,72) | 0,47 (0,31, 0,71) | 0,48 (0,32, 0,73) | 0,49 (0,32, 0,74) | 0,49 (0,32, 0,74) | 0,48 (0,32, 0,73) |
| 8 | 0,46 (0,3, 0,7) | 0,47 (0,31, 0,72) | 0,48 (0,31, 0,73) | 0,47 (0,3, 0,72) | 0,49 (0,32, 0,75) | 0,49 (0,32, 0,75) | 0,49 (0,32, 0,76) | 0,48 (0,31, 0,73) |
| 9 | 0,38 (0,24, 0,6) | 0,39 (0,25, 0,62) | 0,39 (0,25, 0,62) | 0,38 (0,24, 0,61) | 0,4 (0,25, 0,64) | 0,4 (0,25, 0,64) | 0,41 (0,25, 0,65) | 0,39 (0,25, 0,62) |
| 10 | 0,28 (0,17, 0,46) | 0,3 (0,18, 0,49) | 0,3 (0,18, 0,49) | 0,29 (0,18, 0,48) | 0,31 (0,19, 0,51) | 0,31 (0,19, 0,51) | 0,31 (0,19, 0,51) | 0,3 (0,18, 0,49) |
| Civil status |  |  |  |  |  |  |  |  |
| Single | Reference group |  |  |  |  |  |  |  |
| Married/cohabiting | 1,25 (1, 1,56) | 1,27 (1,02, 1,58) | 1,27 (1,02, 1,59) | 1,26 (1,01, 1,57) | 1,27 (1,02, 1,58) | 1,27 (1,02, 1,58) | 1,28 (1,03, 1,59) | 1,26 (1,02, 1,57) |
| Working share |  |  |  |  |  |  |  |  |
| Part-time | Reference group |  |  |  |  |  |  |  |
| Full-time | 0,78 (0,62, 0,98) | 0,81 (0,65, 1,01) | 0,8 (0,64, 1) | 0,8 (0,64, 1,01) | 0,81 (0,65, 1,02) | 0,81 (0,65, 1,02) | 0,82 (0,65, 1,02) | 0,81 (0,65, 1,01) |
| Poor self-rated health | 1,23 (1,13, 1,33) | 1,23 (1,13, 1,33) | 1,22 (1,12, 1,33) | 1,23 (1,13, 1,34) | 1,22 (1,12, 1,33) | 1,23 (1,13, 1,33) | 1,22 (1,12, 1,33) | 1,23 (1,13, 1,33) |
| Sector |  |  |  |  |  |  |  |  |
| Public | Reference group |  |  |  |  |  |  |  |
| Private | 1,28 (1,17, 1,17) | 1,18 (0,92, 1,52) | 1,19 (0,93, 1,53) | 1,18 (0,92, 1,52) | 1,17 (0,91, 1,51) | 1,19 (0,93, 1,53) | 1,16 (0,9, 1,49) | 1,17 (0,91, 1,5) |
| Birth cohort |  |  |  |  |  |  |  |  |
| Born in 1948 or earlier | Reference group |  |  |  |  |  |  |  |
| Born in 1949 or later | 0,98 (0,78, 1,25) | 0,98 (0,77, 1,25) | 0,98 (0,77, 1,24) | 0,97 (0,76, 1,23) | 0,98 (0,77, 1,24) | 0,98 (0,77, 1,24) | 0,98 (0,77, 1,24) | 0,97 (0,77, 1,23) |
| Sector#Later49 | 0,60 (0,42, 0,85) | 0,59 (0,42, 0,84) | 0,59 (0,42, 0,84) | 0,60 (0,42, 0,85) | 0,60 (0,42, 0,85) | 0,59 (0,41, 0,84) | 0,60 (0,42, 0,86) | 0,60 (0,42, 0,85) |
| Note: Job stress, job variety, appreciated, learning opportunities and autonomy are continuous variables on a scale from 1-4. Job resources and job strain are binary variables. | | | | | | | | |


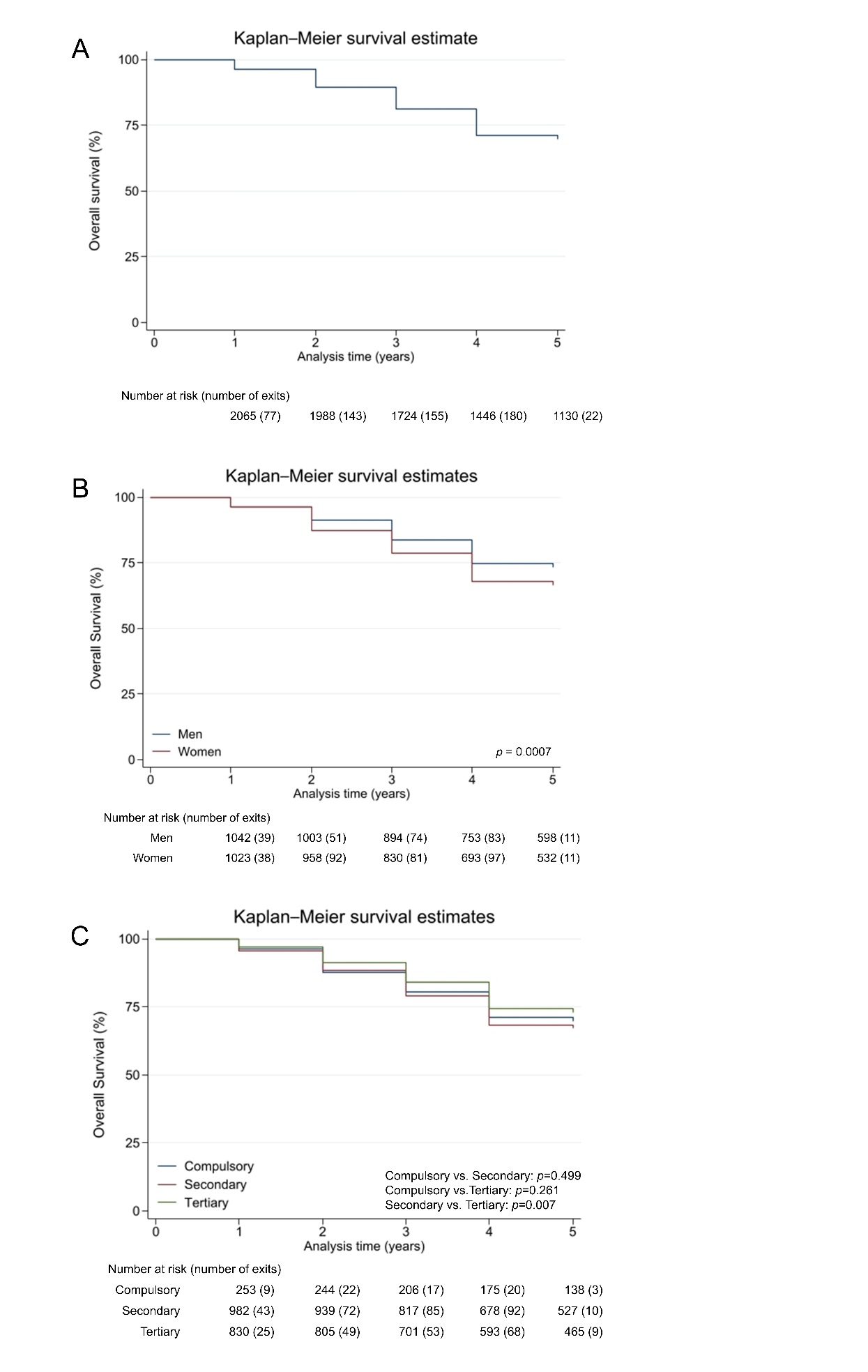


Figure S1. Kaplan-Meier curves for survival analysis in the study cohort and subgroup. A) Overall survival across the entire population. B) Survival across gender. C) Survival across educational attainment.





Figure S2. The mean level of exposure (range 0-3) of the six individual items measuring psychosocial working environment at each age of employment exit (including those who were right-censored).


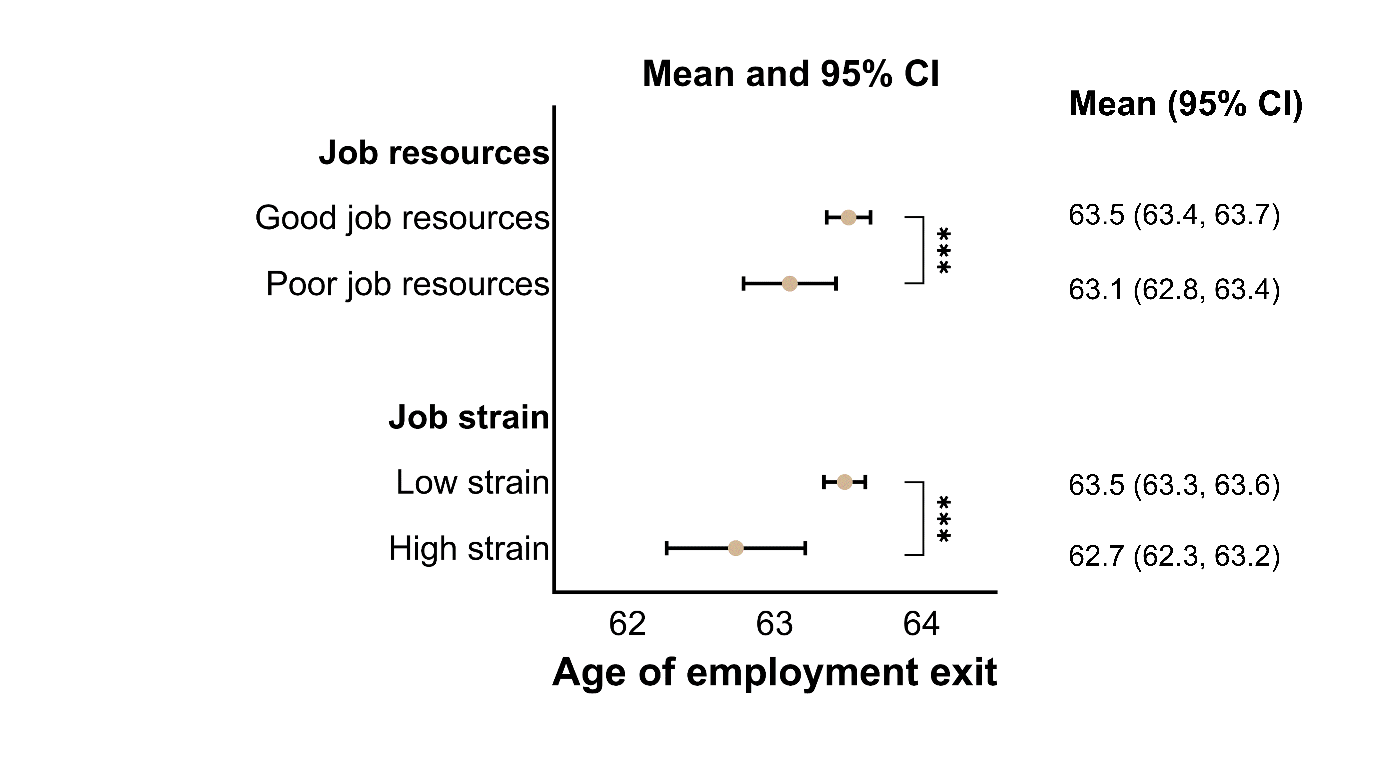


Figure S3. The average age of employment exit over job resources and job strain (n=577).
